# Supportive parent-adolescent relationships as a foundation for adolescent emotion regulation and adjustment

**DOI:** 10.3389/fpsyg.2023.1193449

**Published:** 2023-07-20

**Authors:** Erin L. Ratliff, Amanda S. Morris, Lixian Cui, Jens E. Jespersen, Jennifer S. Silk, Michael M. Criss

**Affiliations:** ^1^Department of Pharmacology and Physiology, Oklahoma State University Center for Health Science, Tulsa, OK, United States; ^2^Department of Psychology, Oklahoma State University, Stillwater, OK, United States; ^3^Division of Arts and Sciences, New York University, Shanghai, China; ^4^Department of Psychiatry and Behavioral Sciences, Oklahoma State University Center for Health Science, Tulsa, OK, United States; ^5^Department of Psychology, University of Pittsburgh, Pittsburgh, PA, United States; ^6^Department of Human Development and Family Science, Oklahoma State University, Tulsa, OK, United States

**Keywords:** parent-adolescent relationship, depressive symptoms, emotion regulation, prosocial behavior, aggressive behavior

## Abstract

**Introduction:**

The purpose of this investigation was to examine the influence of supportive parent-adolescent relationships on adolescent adjustment (i.e., prosocial behavior, aggression, depressive symptoms) both directly and indirectly (*via* adolescent emotion regulation). Scholars have posited that adolescent emotion regulation (ER) may serve as an underlying mechanism in the link between parenting and adolescent adjustment. Supportive parent-adolescent relationships (i.e., openness, acceptance, emotional responsiveness) may be a key emotion socialization mechanism influencing adolescent ER.

**Methods:**

The sample included 206 adolescents (Age Range= 10–18years; 51% female; 70.4% ethnic minorities) and one primary caregiver (83.3% biological mothers, 38.7% single parents). Structural equation modeling (SEM) was implemented to investigate the direct and indirect (*via* adolescent ER) effects of supportive parent-adolescent relationships on adolescent adjustment. We also explored whether these indirect and direct effects varied by adolescent sex and age.

**Results:**

Results suggested direct and indirect (*via* adolescent ER) links between supportive parent-adolescent relationships and adolescent prosocial behavior, aggressive behavior, and depressive symptoms. Moreover, evidence indicated that many of these pathways were significant for boys but not girls. No evidence of youth age as a moderator was found.

**Discussion:**

These findings highlight the important role supportive parent-adolescent relationships play in adolescent emotional and behavioral adjustment. Parenting programs could focus on facilitating a mutually responsive parent-adolescent relationship with a specific focus on the dynamic nature of emotion socialization during adolescence.

## 1. Introduction

Intense and labile emotional experiences are often a key characteristic of adolescence (Silk et al., 2003). The development of emotion regulation (ER), or the ability to recognize and regulate one’s emotions, plays an important role in adolescent adjustment. For example, difficulty regulating negative emotions can influence the development of various forms of adolescent psychopathology including both internalizing and externalizing issues (Heleniak et al., 2016; Compas et al., 2017). Further, a more supportive parent-adolescent relationship may contribute to the development of more effective adolescent ER skills which, in turn, may promote positive adolescent development (e.g., prosocial behavior). In contrast, an emotionally unsupportive parent-adolescent relationship may undermine optimal development of ER skills, resulting in adolescent maladjustment (e.g., internalizing and externalizing issues; Branje et al., 2008; Cui et al., 2020).

Studies of parental influences on child adjustment can be categorized into three conceptual models: (1) parent-driven effects focusing on parent behavior; (2) child-driven effects recognizing the influence of child characteristics on parent behavior; and (3) relationship models based on the notion that parent–child relationships are comprised of more than both the parent’s and child’s behaviors (Laursen and Collins, 2009; Lougheed, 2020). Relationship models capture the dyadic nature of the parent-adolescent relationship and suggest that they are important contexts for socialization during adolescence. Moreover, as the parent–child relationship changes to accommodate increases in adolescent autonomy and decision-making (Lougheed, 2019), examining parent-adolescent interactions at the dyadic-level offers insight into the mechanisms linking parenting behaviors to adolescent adjustment. The current study sought to test both the direct and indirect (*via* adolescent ER) effects of dyadic, supportive parent-adolescent relationships on positive and negative developmental outcomes among youth in a predominantly low-income, single-parent, and ethnic minority sample.

ER provides a critical link to understanding developmental psychopathology during adolescence - a period marked by dramatic increases in mental health issues such as depression and anxiety (Young et al., 2019). Rutherford et al. (2015) postulate that ER plays a role in every aspect of human functioning, including mental and physical health, and the formation and maintenance of relationships with others. Emotion dysregulation, specifically dysregulation of sadness and anger, is a feature of both internalizing and externalizing disorders (Zeman et al., 2002; Beauchaine and Cicchetti, 2019). Some researchers have proposed an emotion specificity hypothesis in which children with externalizing problems may display more anger and have difficulty regulating anger, and others with internalizing problems may display more sadness and have difficulty regulating sadness (Zeman et al., 2002; Te Brinke et al., 2021). Further, adolescents who employ effective ER strategies are more likely to use effective social skills, engage in greater prosocial behaviors, and exhibit fewer problem behaviors (Rutherford et al., 2015).

Morris et al. (2007) developed the tripartite model of ER which posits that ER strategies develop through observation of parents’ own regulation strategies, parental emotion guidance and coaching, and the emotional climate of the family. Studies have documented the associations between emotion-related parenting and child ER development, supporting the notion that the development of ER occurs within the context of the family and more specifically, the parent-adolescent relationship (Morris et al., 2018). For example, Cui et al. (2020) found supportive parent emotion socialization practices predicted increases in ER abilities in low-income adolescent females whereas unsupportive practices predicted greater internalizing issues over time. Importantly, previous studies have found adolescents from low-income families are at a greater risk for ER difficulties (Steinberg et al., 2006; Breslau et al., 2017) with evidence suggesting this may be due in part to parenting styles characterized by high levels of intrusiveness and control (O’Neal and Magai, 2005; Consedine et al., 2012). Thus, investigating the role of the parent-adolescent relationship as an ER context, may be particularly important for understanding adjustment outcomes in this population.

Supportive relationships between parents and adolescents are characterized by openness, acceptance, and emotional responsiveness (Criss et al., 2016). In the following paragraphs, we review findings related to each of these elements of supportive parent-adolescent relationships. Parent–child openness, which concerns both parent and child’s open communication about emotional needs, reflects the degree of warmth and responsiveness in the relationship. Research has shown that parent-adolescent openness and feelings of emotional connectedness were significantly and positively related to prosocial behaviors (Kapetanovic et al., 2019). Similarly, research has demonstrated that parent-adolescent communication styles characterized by high levels of open and clear communication and empathetic responsiveness were related to mutually supportive parent-adolescent relationships (Seiffge-Krenke and Pakalniskiene, 2011) and fewer adolescent ER difficulties and depressive symptoms (Brenning et al., 2015). Acceptance in the parent–child relationship reflects the degree to which parents show supportive, accepting, and emotionally responsive behavior. Parental acceptance is associated with greater psychological adjustment (Dwairy, 2010) and emotional stability (Mendo-Lázaro et al., 2019) in adolescence. Thus, openness and acceptance may support parent-adolescent interactions that are more conducive to communication, adolescent disclosure, and emotional responsiveness (Morris et al., 2018).

The capacity for emotional responsiveness, a component of supportive parent-adolescent relationships, is defined as the awareness and responsivity to another’s emotions during social exchanges (Feldman et al., 2013). Research suggests that these experiences, characterized by equitable give-and-take, are particularly important for social and emotional outcomes during childhood and adolescence. Studies have found that high levels of emotional responsiveness during parent-adolescent interactions were significantly associated with high levels of feelings of closeness to parents and peers (Laursen et al., 2000; Mastrotheodoros et al., 2019) and adolescent dialogical skills (i.e., the capacity for perspective-taking and empathy) during other social interactions (Feldman et al., 2013). Silk et al. (2007) found, among early adolescent boys in low-income families, maternal acceptance and emotional responsivity predicted lower levels of internalizing issues. Notably, the protective effects of the parent-adolescent relationship were attenuated among adolescents with higher exposure to neighborhood risk, suggesting that the buffering effects of family contextual factors may be limited in particularly high-risk environments. In contrast, parent-adolescent interactions lacking emotional responsiveness may contribute to adolescent emotion dysregulation and increased risk for adolescent psychopathology. Studies have shown low levels of parent–child emotional responsiveness are significantly related to high levels of adolescent emotion dysregulation and depressive symptoms (Yap et al., 2010; Van Lissa et al., 2019). Taken together, these findings suggest the three factors comprising supportive parent-adolescent relationships. Openness, acceptance, and emotional responsiveness, may work in tandem to influence adolescent ER and subsequent adjustment outcomes.

Associations between supportive parent-adolescent relationships, ER, and adolescent outcomes may differ by sex and age. Some studies have found the link between parental emotional support and adolescent externalizing symptoms is stronger among girls (de Kemp et al., 2007); whereas other studies have found no sex differences in the link between parental emotional support and acceptance and adolescent externalizing symptoms (Garthe et al., 2018). Moreover, evidence in the literature has demonstrated age differences in the link between parenting and adjustment. For instance, Helsen et al. (2000) found parental emotional support was more strongly related to internalizing symptoms among younger adolescents compared to older youth. Another study found no age differences in the link between parental support and adolescent internalizing symptoms (Meadows et al., 2006). Additional research is needed to further elucidate the moderating role of age and sex among these associations.

Previous studies have explored ER as a mediator in the link between parenting characteristics and adolescent adjustment (e.g., Weissman et al., 2019; Perry et al., 2020). While these studies have added to our understanding of this literature, some gaps remain. First, few studies have focused on adolescent ER as the mechanism linking supportive relationships between parents and adolescents and adolescent adjustment. Further, we examined supportive parent-adolescent relationships as a latent construct using multi-method, multi-informant approaches. The use of observational and self-report data from both parent and adolescent to examine relational constructs such as acceptance, openness, and emotional responsiveness adds to a growing literature that captures the dyadic and increasingly egalitarian interactions between parents and adolescents. Second, there is limited research investigating ER as a mediator in the relationship between indicators of parent-adolescent relationship quality and adjustment outcomes in low-income, single-parent, and ethnic minority samples. Given past research suggesting adolescents from low-income families are at increased risk for ER difficulties (Steinberg et al., 2006; Breslau et al., 2017), it is particularly important to explore how ER may influence these relationships. Lastly, there have been few published investigations examining whether these associations vary by adolescent age and gender. In the current study, we addressed these gaps by first examining direct and indirect (*via* adolescent anger and sadness regulation) links between supportive parent-adolescent relationships and adolescent adjustment (i.e., prosocial behavior, depressive symptoms, and aggressive behavior) in a predominantly low-income, single parent, ethnic minority sample. It was hypothesized that supportive parent-adolescent relationships would be directly and indirectly (*via* adolescent anger and sadness regulation) related to adolescent adjustment. In addition, we explored whether these pathways were moderated by adolescent age and sex. No specific hypotheses regarding sex and age differences were made, as previous research findings related to these factors were inconclusive.

## 2. Materials and methods

### 2.1. Participants

The sample consisted of 206 families with adolescents who participated in the Family and Youth Development Project (FYDP; citation withheld for masked review). The purpose of the FYDP was to examine predictors and outcomes of adolescent ER. Data were collected from urban areas of a southern Midwest region of the United States from both adolescents (*M* age = 13.38 years, SD = 2.32, Age Range = 10–18 years; 51% female; 32% African American, 29.6% European American, 19.4% Latino American, 19% multiple racial/ethnic groups) and their primary caregivers (83.3% biological mothers, 10.7% biological fathers, 2% grandparents, 4% other). The sample was predominantly comprised of low-income (*Median* annual income = $40,000, 47.5% of families were receiving welfare or public assistance) families with an average of 4.35 people living in each home and 38.7% headed by single parents.

### 2.2. Procedures and measures

Families were recruited through fliers and convenience snowball sampling methods. Participants were asked to come to a university laboratory to participate in the study. Following the IRB protocol, the purpose and procedure of the study were explained to adolescents and their primary caregivers before they signed consent and assent forms. Following the assent/consent process, parents and adolescents were separated to complete questionnaires assessing parenting and adjustment. After completing the questionnaires, parents and adolescents were brought together to participate in an emotion-eliciting conflict resolution task which asked dyads to discuss their most frequent conflicts. Interactions were videotaped for later coding. The laboratory assessment lasted 2 h on average. Parents and adolescents received financial compensation for their time spent in the lab.

#### 2.2.1. Parental acceptance

Adolescents completed the parental acceptance scale which assesses the degree to which the parent displays supportive, accepting, and emotionally responsive behavior when interacting with the adolescent (Schaefer, 1965). The parental acceptance scale is a 10-item Likert scale ranging from 1 (*not like her/him*) to 3 (*a lot like her/him*) and includes items such as “My mother/father is a person who makes me feel better talking over my worries with her/him,” and “makes me feel like the most important person in her/his life.” Mean scores were calculated with higher scores indicating greater parental acceptance. Cronbach’s α was 0.92.

#### 2.2.2. Parent-adolescent openness

Parents and adolescents each reported the extent to which the parent and adolescent have a relationship marked by open communication, support, and emotional responsiveness using a 5-point scale ranging from 1 (*Definitely not*) to 5 (*Definitely*). This instrument was adapted from the Student-Teacher Relationship Scale (Pianta, 2001) and the Adult-Child Relationship Scale (Criss et al., 2003) and included 10 items such as “If upset about something, I would talk with my mother/father about it,” and “I liked asking my mother/father about how things were going for her/him.” Wording of the items was adjusted for parent-report to assess parent openness with their adolescent; “If I was upset about something, I would tell my child about it,” and “I was very open about sharing my feelings and telling my child how things were going.” While the ACRS measure focused on parent-to-child behavior, it was modified in the current project to assess both parent-to-adolescent and adolescent-to-parent behavior, making it more of a dyadic measure of parent-adolescent relationship quality. Mean scores were calculated, with higher scores reflecting greater parent-adolescent openness. Cronbach’s αs were 0.92 for adolescent report and 0.84 for parent report.

#### 2.2.3. Observed parent-adolescent relationship quality

Parent-adolescent relationship quality was assessed during the 6-min conflict resolution task which asked dyads to discuss their top 5 most frequent conflicts identified using the modified Conflict Frequency Scale (Melby et al., 1998). Both parents and adolescents completed the 33-item questionnaire prior to the task which consists of possible conflict topics including but not limited to, “Attitude/respect,” “Chores at home,” and “Homework.” Parents and adolescents rated how frequently in the past year they had each conflict on a 5-point Likert scale from “Never” to “Very Often.” The 5 topics rated most frequent by the dyad were selected for use in the conflict resolution task.

Interactions during the task were coded using a revised coding scheme developed originally by Rand Conger and his colleagues (Melby et al., 1998). Research assistants rated the quality of the parent-adolescent relationship on a 9-point scale. A low score indicates an unhappy, emotionally unsatisfying, or brittle relationship and reflects low levels of relationship quality between parent and adolescent. A high score indicates a warm, open, happy, and emotionally responsive relationship and reflects high levels of relationship quality. Evidence of good communication, humor, responsiveness, positive responses to the other’s verbalizations, warmth, and awareness of the other person’s life and daily activities are considered as indicators of good relationship quality. Evidence of hostility, intrusiveness, lecturing or moralizing (usually the parent), constraining verbal expression, inducing guilt, or invalidating feelings are considered indicators of poor relationship quality. Based on 20% of the videos coded twice, interrater reliability for parent-adolescent relationship quality was assessed using intraclass correlations (ρ = 0.71, *p* < 0.001).

#### 2.2.4. Adolescent emotion regulation

Parents and adolescents each reported on adolescents’ abilities to regulate their emotions using the Children’s Emotion Management Scale: Sadness and Anger scales (CSMS; Zeman et al., 2001). The sadness and anger coping subscales were used as indicators of adolescent ER. The sadness coping subscale included five items such as “I try to calmly deal with what is making me sad.” One item (“When I am sad, I do something totally different until I calm down”) was discarded to improve reliability (final Cronbach’s α was 0.61 for adolescent report, and 0.60 for parent report). The anger coping subscale included four items such as “I stay calm and keep cool when I’m feeling mad.” Cronbach’s α was 0.74 for adolescent report, 0.79 for parent report. Wording of these items was modified for parent report of adolescent ER. The scale ranged from 0 (*Not true*) to 2 (*Very true*). Means scores were calculated for both subscales, with higher scores indicating greater emotion coping strategies.

#### 2.2.5. Adolescent prosocial behavior

Parents and adolescents each reported on adolescents’ prosocial behavior during the past year on a 3-point scale from 0 (*Not true*), 1 (*Sometimes*), to 2 (*True*). This measure (from the Strengths and Difficulties Questionnaires, SDQ, Goodman and Scott, 1999) included 5 items such as “I try to be nice to other people,” and “I care about their feelings.” Mean scores were calculated, with higher scores reflecting greater prosocial behavior. Cronbach’s α was 0.81 for adolescent-report and 0.70 for parent-report. A composite score was calculated based on the average (*r* = 0.25, *p* < 0.001) of adolescent and parent ratings.

##### 2.2.6. Adolescent aggressive behavior

Parents and adolescents each reported on adolescents’ aggressive behavior using 14 items from the Problem Behavior Frequency Scale (Farrell et al., 2000), which assessed the frequency of physical, relational, and verbal aggression. Example items include: “Get in a fight in which someone was hit,” “Spread a rumor,” and “Insult someone’s family.” Both parents and adolescents were asked to indicate how frequently the adolescents engaged in each behavior during the past year using the following scale ranging from 1 (*Never*), 2 (*1–2 times*), 3 (*3–4 times*), 4 (*5–6 times*), to 5 (*7 or more times*). Mean scores were calculated, with higher scores reflecting greater aggressive behavior. Cronbach’s α was 0.88 for adolescent report and 0.90 for parent report. The adolescent and parent ratings were averaged (*r* = 0.38, *p* < 0.001) to create the adolescent aggressive behavior composite score.

##### 2.2.7. Adolescent depressive symptoms

Adolescents reported on their own depressive symptoms during the last 2 weeks using the Child Mood & Feelings Questionnaire (MFQ-C, Angold and Costello, 1987) using a 3-point scale ranging from 0 (*Not true*), 1 (*Sometimes*), to 2 (*True*). This measure includes 33 items such as “I felt miserable or unhappy,” and “I thought there was nothing for me in the future.” Cronbach’s α was 0.93 and scores were averaged with higher scores indicating greater depressive symptoms.

#### 3. Results

##### 3.1. Analytical plan

First, mean sex and age group differences on study variables were tested using *t*-tests. To answer our research question, structural equation modeling (SEM) was implemented to test the theoretical models using *M*plus version 6.12 (Muthén and Muthén, 2012). Parental acceptance, both parent and adolescent-reports of parent-adolescent openness and observed parent-adolescent relationship quality were used as four indicators of supportive parent-adolescent relationships. Parent-report and adolescent-report of sadness or anger regulation were two indicators for the sadness or anger regulation latent construct, respectively. Anger and sadness regulation were examined in separate models. Using the classic two-step modeling procedure (Anderson and Gerbing, 1988), measurement models of the latent variables were tested and modified first, followed by the structural models used to test the hypothesized theoretical associations. The chi-square test of fit was supplemented with the comparative fit index (CFI > 0.95) and the root mean square error of approximation (RMSEA <0.06, Hu and Bentler, 1999). Indirect effects of supportive parent-adolescent relationships on outcome variables through anger or sadness regulation were estimated, and bootstrapping was used to estimate the standard errors and 95% bias-corrected confidence intervals of the coefficients in *M*plus. Finally, multi-group analysis in *M*plus was conducted to examine sex and age differences in the associations. A median split was used to create two age groups (ages 10–13, 50.5% vs. 14–18, 49.5%) based on early and middle age ranges of adolescence put forth by the American Academy of Pediatrics (Allen and Waterman, 2019). Factor loadings of the observed variables and variances of the latent factors were constrained to be equal across groups to examine measurement invariance. Next, constraints were placed on all path coefficients in the structural models and individually relaxed based on theory and improvement in model fit based on chi-square difference (Δ*χ*^2^) test.

##### 3.2. Descriptive analyses

Adolescent females reported greater openness with their parents compared to adolescent males, *t* (204) = 3.02, *p* < 0.01 and were rated to have higher parent-adolescent relationship quality than adolescent males, *t* (197) = 2.27, *p* = 0.02. Parents reported adolescent females to have higher scores on anger regulation than males, *t* (202) = 2.18, *p* = 0.03. Both adolescent-reports and parent-reports indicated females had higher levels of prosocial behavior than males, *t* (196.84) = 4.29, *p* < 0.001, and *t* (191.80) = 2.76, *p* < 0.01, respectively. Adolescent males reported slightly more aggressive behavior than females, *t* (204) = −1.99, *p* < 0.05. Depressive symptoms did not differ by sex, *t* (203) = −0.69, *p* = 0.49.

Younger adolescents reported greater parental acceptance, and openness than older adolescents, *t* (199.59) = 3.53, *p* = 0.001, and *t* (204) = 2.28, *p* = 0.02, respectively. Younger adolescents also were rated to have higher relationship quality with parents, *t* (197) = 2.22, *p* = 0.03, and rated by parents to have higher levels of openness and prosocial behavior, *t* (202) = 2.38, *p* = 0.02, and *t* (202) = 2.24, *p* = 0.03, respectively. Younger adolescents reported marginally fewer depressive symptoms, *t* (203) = −1.86, *p* = 0.06. No age differences in ER or aggressive behavior were found. Descriptive statistics for all variables for the full sample and by age and sex can be found in Table 1. Correlations among all study variables are presented in Table 2.

**Table 1:** Descriptive statistics.

Variables	Full Sample (*N* = 206)	Females (*n* = 105)	Males (*n* = 101)	Younger (*n* = 104)	Older (*n* = 102)
Adolescent age	13.38 (2.32)	13.39 (2.36)	13.37 (2.29)	11.37 (1.08)	15.44 (1.06)
Adolescent sex	51% female	–	–	50% female	52% female
Supportive Parent-Adolescent Relationship					
Parental Acceptance (A)	2.51 (0.51)	2.53 (0.52)	2.48 (0.49)	2.63 (0.46)	2.38 (0.52)
Relationship Quality (O)	4.71 (2.63)	5.12 (2.70)	4.28 (2.53)	5.11 (2.64)	4.29 (2.59)
Openness (A)	3.61 (0.97)	3.81 (0.99)	3.41 (0.91)	3.76 (0.92)	3.46 (1.00)
Openness (P)	4.07 (0.63)	4.11 (0.61)	4.02 (0.66)	4.17 (0.56)	3.96 (0.69)
Emotion Regulation					
Anger Regulation (A)	1.19 (0.52)	1.20 (0.52)	1.18 (0.52)	1.21 (0.54)	1.17 (0.50)
Sadness Regulation (A)	1.32 (0.49)	1.33 (0.48)	1.30 (0.50)	1.29 (0.52)	1.34 (0.45)
Anger Regulation (P)	1.00 (0.51)	1.07 (0.48)	0.91 (0.52)	1.01 (0.49)	0.98 (0.52)
Sadness Regulation (P)	1.08 (44)	1.05 (0.44)	1.11 (0.44)	1.06 (0.44)	1.09 (0.45)
Outcome Variables					
Prosocial (A)	1.57 (0.43)	1.69 (0.38)	1.45 (0.44)	1.61 (0.43)	1.53 (0.43)
Prosocial (P)	1.63 (0.36)	1.70 (0.32)	1.56 (0.38)	1.69 (0.33)	1.58 (0.38)
Depressive (A)	0.37 (0.33)	0.36 (0.34)	0.39 (0.33)	0.33 (0.30)	0.42 (0.36)
Aggression (A)	1.43 (0.52)	1.36 (0.50)	1.51 (0.53)	1.37 (0.51)	1.50 (0.52)
Aggression (P)	1.55 (0.62)	1.49 (0.53)	1.61 (0.71)	1.55 (0.60)	1.55 (0.65)

Means and standard deviations (in parentheses); A, adolescent reports; P, parent reports; O, observer rating.

**Table 2:** Bivariate correlations.

*Variables*	*1*	*2*	*3*	*4*	*5*	*6*	*7*	*8*	*9*	*10*	*11*	*12*	*13*	*14*	*15*
1. Adolescent age	*–*														
2. Adolescent sex^a^	−0.01	*–*													
S-PAR															
3. Parental acceptance (A)	−0.26^***^	−0.06	*–*												
4. Relationship quality (O)	−0.15^*^	−0.16^*^	0.35^***^	*–*											
5. Openness (A)	−0.20^**^	−0.21^**^	0.63^***^	0.28^***^	*–*										
6. Openness (P)	−0.12	−0.07	0.43^***^	0.29^***^	0.39^***^	*–*									
Emotion regulation															
7. AR (A)	−0.03	−0.02	0.24^**^	0.29^***^	0.30^***^	0.28^***^	*–*								
8. SR (A)	0.05	−0.03	0.18^*^	0.10	0.12	0.15^*^	0.42^***^	*–*							
9. AR (P)	−0.04	−0.15^*^	0.17^*^	0.23^**^	0.19^**^	0.21^**^	0.36^***^	0.18^*^	*–*						
10. SR (P)	0.03	0.08	0.18^*^	0.18^*^	0.13	0.21^**^	0.30^***^	0.25^***^	0.57^***^	*–*					
Outcome variables															
11. Prosocial (A)	−0.06	−0.29^***^	0.36^***^	0.17^*^	0.40^***^	0.31^***^	0.45^***^	0.30^***^	0.24^***^	0.21^**^	*–*				
12. Prosocial (P)	−0.15^*^	−0.19^**^	0.25^***^	0.32^***^	0.14^*^	0.40^***^	0.28^***^	0.19^**^	0.31^***^	0.31^***^	0.25^***^	*–*			
13. Depressive symptoms (A)	0.15^*^	0.05	−0.23^**^	−0.22^**^	−0.24^**^	−0.09	−0.34^***^	−0.14^*^	−0.22^**^	−0.16^*^	−0.03	−0.19^**^	*–*		
14. Aggression (A)	0.18^**^	0.14^*^	−0.29^***^	−0.32^***^	−0.22^**^	−0.24^**^	−0.43^***^	−0.20^**^	−0.34^***^	−0.18^*^	−0.28^***^	−0.29^***^	0.42^***^	*–*	
15. Aggression (P)	0.03	0.09	−0.23^**^	−0.27^***^	−0.13	−0.41^***^	−0.34^***^	−0.21^**^	−0.43^***^	−0.36^***^	−0.25^***^	−0.48^***^	0.17^*^	0.38^***^	*–*

S-PAR, Supportive Parent-adolescent Relationship; (A), adolescent report; (P), parent report; O, observer rating; AR, Anger regulation; SR, Sadness regulation. ^*^ *p* < 0.05. ^**^ *p* < 0.01. ^***^ *p* < 0.001.

a Youth sex was coded as 0 (females) and 1 (males).

##### 3.3. Indirect effect models for the full sample

###### 3.3.1. Anger regulation model

**Figure 1:**
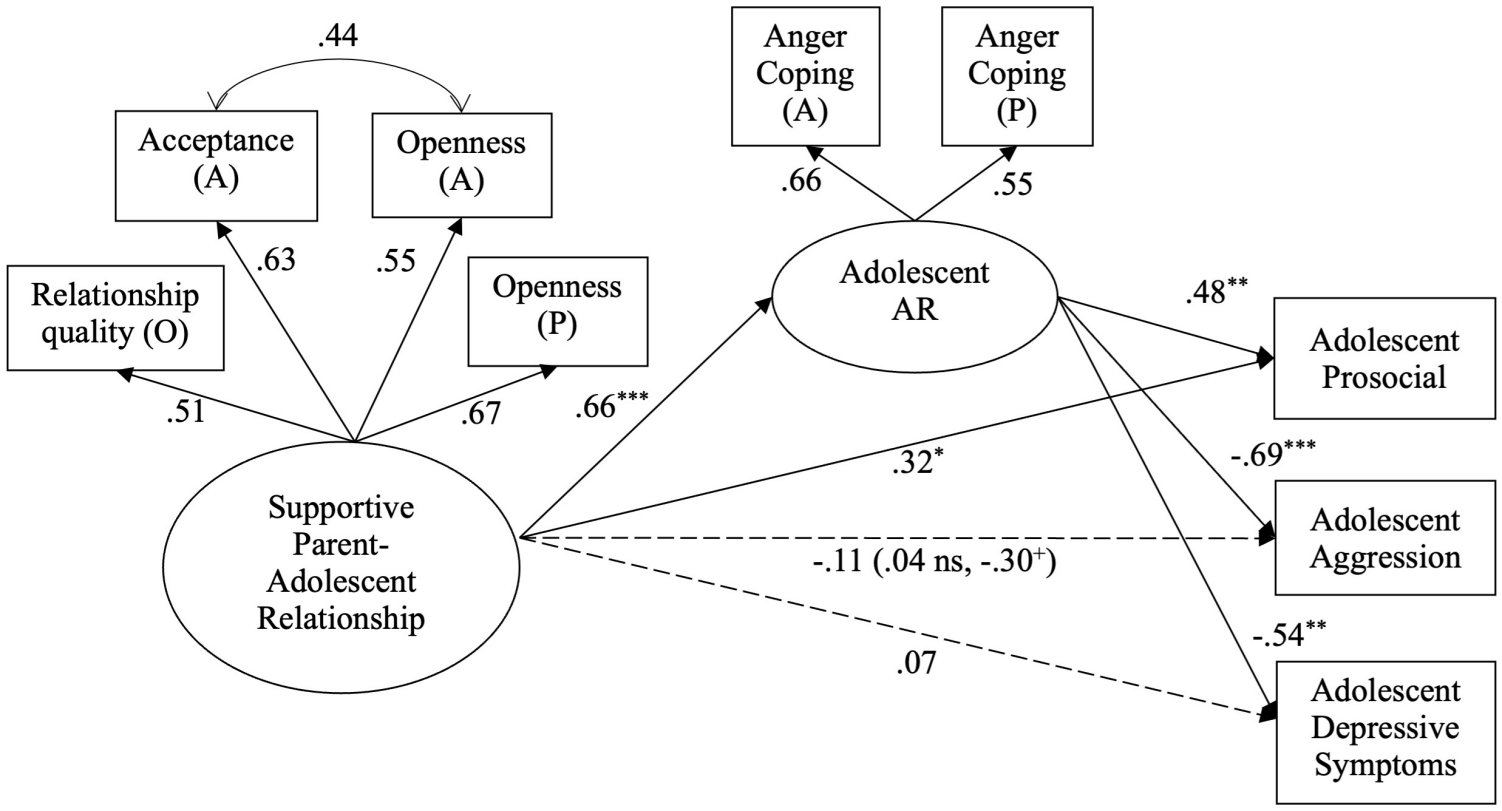
The indirect effects of supportive parent-adolescent relationship on adjustment through anger regulation (AR). All estimates are standardized coefficients. Coefficients for both sex groups are in parenthesis, with females’ coefficient estimate followed by males’. ^+^ *p* < 0.10. ^*^ *p* < 0.05. ^**^ *p* < 0.01. A, adolescent reports; P, parent reports; O, observational.

The measurement model was tested first. Based on modification indices, adolescent report of openness and parental acceptance were permitted to correlate with each other. The final measurement model fit the data well, *χ*^2^ (7) = 7.05, *p* = 0.42; CFI = 1.00; RMSEA = 0.01. Analysis of the structural model fit the data very well, *χ*^2^ (19) = 32.15, *p* = 0.03; CFI = 0.97; RMSEA = 0.06. Supportive parent-adolescent relationships were positively associated with anger regulation, which in turn was positively associated with adolescent prosocial behavior and negatively associated with adolescent aggressive behavior and depressive symptoms (Figure 1). The direct link between supportive parent-adolescent relationships and prosocial behavior was significant. All indirect effects of supportive parent-adolescent relationships on the three adjustment outcomes through anger regulation were significant (Table 3). Thus, supportive parent-adolescent relationships were directly and indirectly related to adolescent prosocial behavior; in contrast, supportive parent-adolescent relationships were indirectly (but not directly) related to adolescent aggression and depressive symptoms.

**Table 3:** Bootstrap tests of indirect effect of supportive parent-adolescent relationship through emotion regulation.

	Prosocial behavior		Aggressive behavior		Depressive symptoms	
	Estimate	95% CI	Estimate	95% CI	Estimate	95% CI
Full Sample						
S-PAR *via* AR:						
Direct effects	0.31	[−0.07, 0.69]	−0.16	[−0.69, 0.72]	0.08	[−0.26, 0.83]
Indirect effects	0.31^**^	[0.14, 0.96]	−0.69^**^	[−2.02, −0.35]	−0.38^**^	[−1.33, −0.15]
S-PAR *via* SR:						
Direct effects	0.41	[−0.04, 0.71]	−0.51	[−1.00, 0.22]	−0.18	[−0.49, 0.18]
Indirect effects	0.21^**^	[0.07, 0.79]	−0.32^**^	[−1.41, −0.09]	−0.12	[−0.54, 0.04]
Girls						
S-PAR *via* AR:						
Direct effects	0.21	[−0.26, 0.49]	0.06	[−0.53, 0.83]	0.01	[−0.30, 0.63]
Indirect effects	0.34^**^	[0.17, 0.87]	−0.60^**^	[−1.74, −0.29]	−0.32^**^	[−1.18, −0.12]
S-PAR *via* SR:						
Direct effects	0.32	[−0.04, 0.54]	−0.29	[−0.75, 0.27]	−0.19	[−0.52, 0.14]
Indirect effects	0.12	[−0.02, 0.65]	−0.13	[−0.83, 0.01]	−0.05	[−0.54, 0.02]
Boys						
S-PAR *via* AR:						
Direct effects	0.21	[−0.26, 0.49]	−0.47	[−1.14, 0.36]	0.01	[−0.30, 0.63]
Indirect effects	0.34^**^	[0.17, 0.87]	−0.60^**^	[−1.74, −0.29]	−0.32^**^	[−1.18, −0.12]
S-PAR *via* SR:						
Direct effects	0.32	[−0.04, 0.54]	−0.81^*^	[−1.48, −0.14]	−0.19	[−0.52, 0.14]
Indirect effects	0.35^**^	[0.15, 0.79]	−0.39^*^	[−1.19, −0.07]	−0.14	[−0.57, 0.10]

S-PAR, Supportive Parent-adolescent Relationship; AR, Anger regulation; SR, Sadness regulation; CI, Confidence interval. ^*^ *p* < 0.05. ^**^ *p* < 0.01. All estimates were unstandardized coefficients.

###### 3.3.2. Sadness regulation model

**Figure 2:**
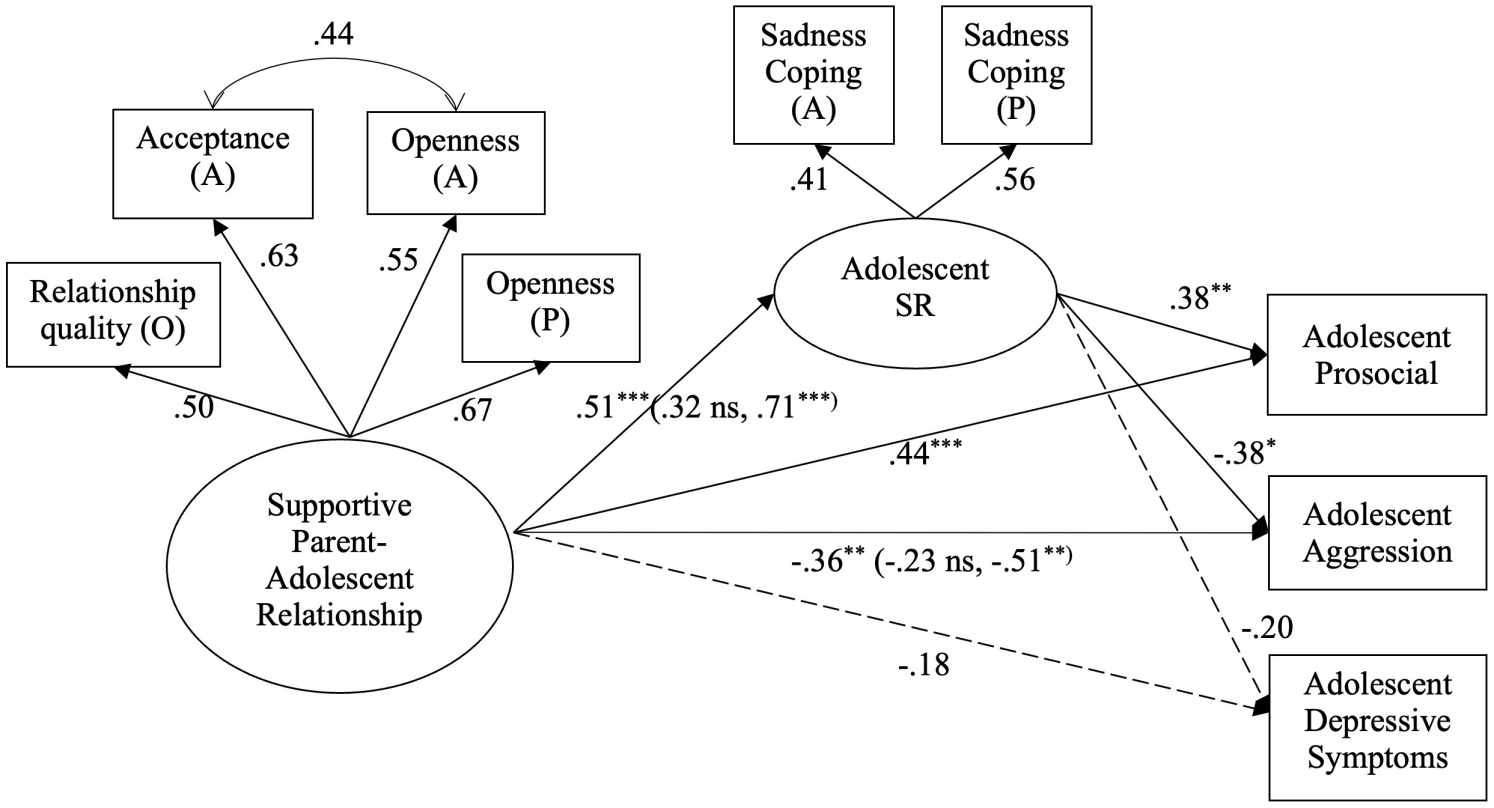
The indirect effects of supportive parent-adolescent relationship on adjustment through sadness regulation (SR). All estimates are standardized coefficients. Coefficients for both sex groups are in parentheses, with females’ coefficient estimates followed by males’. ^*^ *p* < 0.05. ^**^ *p* < 0.01. A, adolescent reports; P, parent reports; O = observational.

The final measurement model fit the data well, *χ*^2^ (7) = 3.41, *p* = 0.84; CFI = 1.00; RMSEA = 0.00. Analysis of the structural model fit the data very well, *χ*^2^ (19) = 25.01, *p* = 0.16; CFI = 0.98; RMSEA = 0.04. Supportive parent-adolescent relationships were positively associated with sadness regulation, which in turn was positively associated with adolescent prosocial behavior and negatively associated with adolescent aggressive behavior but not significantly associated with depressive symptoms (Figure 2). The direct links between supportive parent-adolescent relationships and prosocial behavior and aggression were significant. The indirect effects of supportive parent-adolescent relationships on adolescent prosocial and aggressive behavior through sadness regulation were significant, while the indirect effect on depressive symptoms through sadness regulation was not significant (Table 3). Thus, supportive parent-adolescent relationships were directly and indirectly related to adolescent prosocial behavior and aggression, but supportive parent-adolescent relationships were not significantly related (directly or indirectly) to adolescent depressive symptoms in this model.

##### 3.4. Moderation by adolescent sex

The multi-group measurement model was fit to the data first. The modification indices suggested that only the intercepts for parental acceptance and adolescent report of openness varied by sex, i.e., the intercept of parental acceptance was slightly higher among males, and the intercept of openness was higher among females. The final measurement model fit the data well, *χ*^2^ (29) = 26.01, *p* = 0.63; CFI = 1.00; RMSEA = 0.00 for anger regulation model, and *χ*^2^ (29) = 20.92, *p* = 0.86; CFI = 1.00; RMSEA = 0.00 for sadness regulation model.

###### 3.4.1. Anger regulation model

All the path coefficients in the structural model were then constrained to be equal across sexes. The modification indices suggested that the link between supportive parent-adolescent relationships and adolescent aggressive behavior should be freely estimated, and the new model fit the data better than the fully constrained model, Δ*χ*^2^ (1) = 7.35, *p* < 0.01, with the model fit, *χ*^2^ (59) = 80.69, *p* = 0.03; CFI = 0.96; RMSEA = 0.06. Results suggested that the association between supportive parent-adolescent relationships and adolescent aggressive behavior was marginally significant for males and was not significant for females (Figure 1).

###### 3.4.2. Sadness regulation model

All of the path coefficients in the structural model were constrained to be equal across sexes. The modification indices suggested that the link between supportive parent-adolescent relationships and adolescent aggressive behavior should be freely estimated, and the new model fit the data better than the fully constrained model, Δ*χ*^2^ (1) = 8.45, *p* < 0.01. After releasing this link, the modification indices for the new model suggested that the link between supportive parent-adolescent relationships and sadness regulation should also be freely estimated, and the final model fit the data better, *χ*^2^ (58) = 60.13, *p* = 0.40; CFI = 1.00; RMSEA = 0.02, than the previous model, Δ*χ*^2^ (1) = 6.50, *p* < 0.05. The results suggested the association between supportive parent-adolescent relationships and adolescent sadness regulation was significant for males but not for females, and the association between supportive parent-adolescent relationships and aggressive behavior was also significant for males but not for females. Bootstrapping results showed that the indirect effects of supportive parent-adolescent relationships on prosocial and aggressive behavior were only significant for males (Table 3). In other words, supportive parent-adolescent relationships were directly and indirectly related to aggression for males but not females. None of the indirect effects were significant for females due to the non-significant link between supportive parent-adolescent relationships and sadness regulation.

##### 3.5. Moderation by adolescent age

The multi-group measurement models fit the data well, *χ*^2^ (31) = 28.79, *p* = 0.58; CFI = 1.00; RMSEA = 0.00 for anger regulation model, and *χ*^2^ (31) = 20.48, *p* = 0.93; CFI = 1.00; RMSEA = 0.00 for sadness regulation model. All the path coefficients in the structural model were constrained to be equal at first. The fully constrained models fit the data well, *χ*^2^ (62) = 67.28, *p* = 0.30; CFI = 0.99; RMSEA = 0.03 for anger model, and *χ*^2^ (62) = 50.71, *p* = 0.85; CFI = 1.00; RMSEA = 0.00 for sadness model. The modification indices did not show meaningful constraint releasing suggestions. Thus, there were no significant age differences among the links.

#### 4. Discussion

The current study extended the literature by examining direct and indirect (*via* adolescent sadness and anger regulation) links between supportive parent-adolescent relationships and adolescent adjustment (i.e., prosocial behavior, aggression, depressive symptoms) using a predominantly low-income, single-parent, and ethnic minority sample. Results suggested that ER may serve as one mechanism underlying the association between supportive parent-adolescent relationships and adolescent adjustment outcomes. Specifically, as hypothesized, this study found the effects of supportive parent-adolescent relationships on adolescent depression, aggression, and prosocial behavior were indirect through adolescent ER. Further, we found direct links between supportive parent-adolescent relationships and adolescent prosocial behavior and aggression but not depression. The analyses also showed that adolescent sex (but not age) served as a moderator in some of the pathways.

Parent-adolescent relationships characterized by high levels of parental acceptance and parent-adolescent openness and emotional responsiveness during interactions may help facilitate self-regulatory skills. Providing an environment for these self-regulatory skills to develop may lead to communion (connectedness or relatedness) and agency (independence or self-determination) in children and adolescents (Baumrind, 2013) while also fostering optimal cognitive and social competencies (Criss et al., 2003). Parent-adolescent relationships with high levels of emotional support may be more conducive to adolescent negative emotional expressions facilitating opportunities for supportive emotion socialization behaviors (Morris et al., 2007). Together, the two models examined demonstrated the critical role supportive parent-adolescent relationships may play as an independent factor in the development of adolescent ER and adjustment outcomes for youth at-risk.

##### 4.1. Anger regulation

In support of the first hypothesis, results of the anger model revealed significant indirect effects, such that greater supportive parent-adolescent relationships were associated with greater adolescent anger regulation, which in turn was related to greater prosocial behaviors and less aggression and depressive symptoms. These findings are consistent with Criss et al. (2016) research which found mutual emotional support, represented by parent-adolescent acceptance and openness, to be both directly related to adolescent anger regulation and indirectly related through emotion coaching. Moreover, additional studies have found parental support, emotion coaching, and family cohesion to be positively related to adolescent anger regulation (e.g., Shortt et al., 2010; Hale et al., 2023), suggesting active emotion socialization efforts continue to play a critical role in adolescence.

Studies examining adolescent anger regulation as a predictor of adolescent outcomes have demonstrated that high levels of anger regulation were significantly related to low levels of adolescent externalizing and internalizing behaviors (Shortt et al., 2010; Otterpohl et al., 2021). In the current study, we found similar patterns in that anger regulation was significantly and negatively related to depressive symptoms while sadness regulation was not. This is consistent with prior longitudinal research indicating anger dysregulation, but not sadness dysregulation, was associated with increased depressive symptoms in a sample of low-income, ethnic minority youth (Folk et al., 2014). However, Rueth et al. (2017) found that adolescent adaptive anger regulation mediated the relationship between parent autonomy support and adolescent externalizing and prosocial behaviors, but not internalizing behaviors. Our findings suggest that anger regulation strategies, developed in the context of emotionally responsive parent-adolescent interactions, may be associated with fewer depressive symptoms, possibly by deterring anger suppression strategies which have been shown to increase adolescent internalizing symptoms (Rueth et al., 2017). Indeed, O’Neal et al. (2017) found parent discouragement of their child’s anger significantly predicted increased depressive symptoms during adolescence in a low-income, ethnic minority sample. Results regarding prosocial behavior are consistent with past findings suggesting the ability to regulate one’s expression of anger may be associated with prosocial behavior including empathetic concern, perspective-taking, and cooperation during social interactions (Feldman et al., 2013).

Building upon past research, our findings suggest that adolescent anger regulation is an important mechanism through which the latent construct of supportive parent-adolescent relationships is linked to adolescent prosocial behavior, aggression, and depressive symptoms. In relationships characterized by high levels of acceptance and emotional support, parents may be more likely to validate adolescent’s negative emotional expressions (specifically anger) and consider these opportunities for supportive emotion socialization practices, such as problem-solving and social support (Criss et al., 2016). In turn, adolescents may be more likely to express, rather than suppress, anger in balanced, emotionally responsive parent-adolescent interactions.

In addition to these indirect effects, a significant direct effect was found between supportive parent-adolescent relationships and adolescent prosocial behaviors. This is consistent with past research that showed positive, balanced parent-adolescent interactions were positively related to adolescent prosocial behavior (Feldman et al., 2013). Specifically, emotionally supportive relationships may facilitate empathy development and perspective-taking in adolescents through both their perceptions of parent support as well as their emotional support of their parent (Boele et al., 2019). Notably, there was not a direct effect from supportive parent-adolescent relationships to adolescent aggression or depressive symptoms suggesting adolescent anger regulation may be a critical mechanism linking these associations.

##### 4.2. Sadness regulation

Next, we examined whether the links between supportive parent-adolescent relationships and adolescent adjustment were mediated by sadness regulation. Results of the second model revealed significant indirect effects, such that supportive parent-adolescent relationships were positively and significantly associated with adolescent sadness regulation, which in turn was significantly related to prosocial behavior and aggression in expected directions. However, no indirect effect was found in the pathway to adolescent depressive symptoms. Interestingly, our findings do not support the aforementioned emotion specificity hypothesis, as we found anger regulation was associated with depressive symptoms and aggression, whereas sadness regulation was associated with aggression but not depressive symptoms. However, our findings are consistent with prior research suggesting anger regulation is related to both internalizing and externalizing symptoms (Shortt et al., 2010; Otterpohl et al., 2021). As mentioned previously, Folk et al. (2014) found anger dysregulation, but not sadness dysregulation, was associated with greater depressive symptoms in a sample of low-income, ethnic minority youth. While this may seem counterintuitive, across low-income samples, anger regulation is consistently associated with internalizing symptoms above and beyond sadness regulation (e.g., Folk et al., 2014; O’Neal et al., 2017). Moreover, previous research has noted youth difficulties when reporting on sadness regulation (O’Neal et al., 2017) which may contribute to this unexpected finding. It is also possible adolescents may experience more anger than sadness due to environmental factors such as neighborhood disadvantage (Sullivan et al., 2010). Considering the results of our moderation analyses (discussed below), these associations may vary based on sex.

Similar to the first model, a significant direct effect was found between supportive parent-adolescent relationships and greater adolescent prosocial behavior. A significant direct effect was found between greater supportive parent-adolescent relationships and less adolescent aggression; however, this association only remained significant for males following moderation analyses. Overall, anger regulation appeared to play a larger role in the links between supportive parent-adolescent relationships and adjustment outcomes compared to sadness regulation. Perhaps in the context of a low-income sample, parents may focus their energy on facilitating anger regulation given the implications of ineffective anger regulation at home, in one’s neighborhood, or at school (Sullivan et al., 2010).

##### 4.3. Sex and age differences

To explore our second research question, we examined whether sex and age moderated the links between supportive parent-adolescent relationships and adolescent adjustment outcomes. In regard to the first model (anger regulation), we found the effect of sex resulted in no changes in the findings aside from a marginally significant direct effect between supportive parent-adolescent relationships and aggression for males compared to a nonsignificant effect for females. Past research suggests parent-adolescent support and openness is associated with less aggressive behaviors for both males and females (Branje et al., 2008), however, in the current study, the inclusion of adolescent ER provides a more nuanced understanding of these associations and points to potential differences based on adolescent sex.

In regard to the second model (sadness regulation), after examining the influence of adolescent sex, the link between supportive parent-adolescent relationships and adolescent sadness regulation remained significant for adolescent males only. Further, the association between supportive parent-adolescent relationships and adolescent aggression remained significant for males only as well. Considering socialization pressures that discourage displays of sadness in boys compared to girls (Zeman et al., 2019), it may be that parent-adolescent relationships characterized by high levels of acceptance, openness, and emotional support offer a safe space for boys to express sadness. Moreover, research suggests parental discouragement or invalidation of expressions of sadness in young boys has been shown to contribute to later externalizing symptoms (Poon et al., 2017). These findings suggest supportive parent-adolescent relationships may be more conducive to the development of sadness regulation and aggressive behavior in males compared to females. However, it should be noted that estimates of reliability for the sadness regulation subscales were comparatively low (Cronbach’s α was 0.61 for adolescent report, and 0.60 for parent report) compared to estimates for the anger regulation subscale, thus findings in model 2 should be interpreted with caution.

##### 4.4. Strengths and limitations

There were a number of strengths reflected in this study. The current study contributed to our understanding of the role of supportive parent-adolescent relationships and ER in adolescent adjustment. With a focus on the parent-adolescent relationship and the emotional exchanges within that relationship, we used an observational measure of relationship quality as an indicator of emotional responsiveness in the parent-adolescent relationship. The other indicator of support, openness in communication, as well as measures of adolescent prosocial behavior and externalizing symptoms were also based on both parent and adolescent reports. Because relationship models encompass the inherently dyadic nature of the parent-adolescent relationship, they are more effective than either just examining parent-driven or child-driven models (Laursen and Collins, 2009). As such, utilizing a multi-method approach and multiple informants strengthened the measure by a means advocated by parenting researchers. Moreover, this study recruited a predominantly low-income, single-parent, and ethnic minority sample which strengthens our understanding of the pathways linking parenting to adolescent adjustment among families from disadvantaged neighborhoods. Lastly, we explored the potential moderating effects of both adolescent sex and age which increases our understanding of how adolescent characteristics may influence the findings.

Despite the mentioned strengths, one limitation of the investigation was the cross-sectional design which limited the ability to determine causality or examine change in adolescent adjustment. Future research would benefit from the use of longitudinal designs. It is possible and likely that a bidirectional relationship exists between variables. Adolescents with less depressive symptoms and aggressive behavior, or greater prosocial behaviors may be better at regulating their emotions, which in turn improves interactions with parents. Further, a reciprocal relationship may exist between adolescent adjustment variables and supportive parent-adolescent relationships indicators given our knowledge of the influence of adolescent psychopathology on parenting behaviors (Zvara et al., 2018). While we consider our multi-informant approach a strength, we recognize the parent- and adolescent-reports of adolescents’ aggressive and prosocial behaviors are only modestly correlated with each other. Our decision to use parent, youth, and observer ratings was influenced by research that emphasizes the multi-informant approach to provide a broader perspective on the parent-adolescent relationship as well as our desire to limit the number of analyses rather than running separate analyses for parent and adolescent reports. Another limitation was the low percentage of fathers included in the study. Including a larger percentage of fathers would help us to better understand how parent’s sex may influence indicators of supportive parent-adolescent relationships. Previous research has found differences in emotion socialization behaviors between mother-adolescent and father-adolescent dyads (Poon et al., 2017; Zvara et al., 2018) which is likely to influence the parent-adolescent relationship. Future research should take into consideration the potential effect of different dyad types (e.g., mother-daughter, father-daughter, mother-son, etc.) on supportive parent-adolescent relationships and adolescent adjustment outcomes. Lastly, although we consider our diverse population a strength, it is possible that different patterns of findings would be found in predominantly middle-class, European American, and married samples.

##### 4.5. Implications and conclusions

This study highlights the importance of parent-adolescent relationships characterized by high levels of acceptance, openness, and emotional support as a foundation for supportive emotion socialization strategies in promoting positive adolescent outcomes in a sample of adolescents at-risk. Moreover, this study provides greater insight into the dyadic nature of supportive parent-adolescent relationships, which may have important implications for targeted prevention and intervention programs. Our findings extend the literature by increasing our understanding of the role of parent-adolescent relationships in which both members of the dyad engage in emotionally responsive, open communication and how these interactions may relate to both ER skills and adolescent outcomes in a low-income, ethnic minority sample. Relationship models represent more than the sum of parent-driven effects and child-driven effects and acknowledge the dyadic aspect of the parent–child relationship in which these skills develop. Taking these findings into consideration, parenting programs could focus on facilitating a mutually responsive parent-adolescent relationship with a specific focus on the dynamic nature of emotion socialization during adolescence.

## Data availability statement

The raw data supporting the conclusions of this article will be made available by the authors, without undue reservation.

## Ethics statement

The studies involving human participants were reviewed and approved by the Oklahoma State University Institutional Review Board. Written informed consent to participate in this study was provided by the participants’ legal guardian/next of kin.

## Author contributions

ER, AM, LC, JJ, JS, and MC contributed to the interpretation and application of results. AM, LC, and MC contributed conception and design of the study and data collection and management. AM, LC, JS, and MC contributed to data analysis. LC and ER wrote the first draft of the manuscript. All authors contributed to the article and approved the submitted version.

## Funding

This research was supported by a United States Department of Agriculture, Oklahoma Agriculture Experiment Station Project grant (AB-1-13921), the Oklahoma Center for the Advancement of Science and Technology (OCAST) grant (AA-5-40772 and AA-5-45433; Project # HR11-130), and a National Institute of Child Health and Human Development (AA-5-43382 and 1R15HD072463–01) R15 grant.

## Conflict of interest

The authors declare that the research was conducted in the absence of any commercial or financial relationships that could be construed as a potential conflict of interest.

## Publisher’s note

All claims expressed in this article are solely those of the authors and do not necessarily represent those of their affiliated organizations, or those of the publisher, the editors and the reviewers. Any product that may be evaluated in this article, or claim that may be made by its manufacturer, is not guaranteed or endorsed by the publisher.
